# Diffusivity alterations related to cognitive performance and phenylalanine levels in early-treated adults with phenylketonuria

**DOI:** 10.1186/s11689-025-09622-8

**Published:** 2025-07-02

**Authors:** Jèssica Pardo, Clara Capdevila-Lacasa, Bàrbara Segura, Adriana Pané, Pedro J. Moreno, Glòria Garrabou, Josep M. Grau-Junyent, Carme Junqué, Consortium PKU.cat, Ana Argudo-Ramírez, Judith Cantó, Francesc Cardellach, Climent Casals-Pascual, Gemma Chiva-Blanch, Maria de Talló Forga-Visa, Francesc Josep García-García, Judit García-Villoria, José Manuel González de Aledo-Castillo, Mariona Guitart-Mampel, Paula Isern, Amanda Jiménez, Berta Laudo, Félix Andújar-Sánchez, Rosa Maria López-Galera, José Cesar Milisenda, Cristina Montserrat-Carbonell, Blai Morales, Pedro Juan Moreno-Lozano, Julián Moreno, Mònica Nos, Montserrat Ortega, Emilio Ortega, Joan Padrosa, Abraham José Paredes, Elisa Rubio, Ester Tobías, Josep Torremade, Laura Valls, Roser Ventura, Andrea Vergara-Gómez, Judith Viaplana, Clara Viñals, Jaume Campistol, Dolores García-Arenas, Silvia Meavilla, Aida Ormazabal, Blanca Barrau-Martínez, Arnau González-Rodríguez, Rafael Llorach, Mireia Urpi-Sarda

**Affiliations:** 1 Medical Psychology Unit, Department of Medicine, Institute of Neurosciences, University of Barcelona, C/Casanova 143 (08036) , Barcelona, Catalonia Spain; 2 Fundació de Recerca Clínic Barcelona-Institut d’Investigacions Biomèdiques August Pi i Sunyer (FRCB-IDIBAPS), Barcelona, Catalonia Spain; 3 Biomedical Research Networking Center on Neurodegenerative Diseases (CIBERNED: CB06/05/0018-ISCIII), Barcelona, Catalonia Spain; 4 Biomedical Research Networking Center on Physiopathology of Obesity and Nutrition (CIBEROBN), Barcelona, Catalonia Spain; 5 Endocrinology and Nutrition Department, Muscular and Inherited Metabolic Disorders Adults Unit, Hospital Clínic de Barcelona, Barcelona, Catalonia Spain; 6 Muscular and Inherited Metabolic Disorders Adults Unit, Hospital Clínic de Barcelona, Barcelona, Catalonia Spain; 7 Inherited Metabolic Diseases and Muscle Disorders Research, Centre de Recerca Biomèdica CELLEX – Institut d’Investigacions Biomèdiques August Pi i Sunyer (IDIBAPS) and Faculty of Medicine and Health Sciences, University of Barcelona, Barcelona, Spain; 8 Biomedical Research Networking Center on Rare Diseases (CIBERER), Barcelona, Catalonia Spain

**Keywords:** Phenylketonuria, Neuropsychological performance, Diffusion tensor imaging, Cerebral white matter, Dietary control

## Abstract

**Background:**

Altered white matter (WM) is consistently reported in patients with phenylketonuria (PKU). However, the knowledge about WM microstructural integrity in early-treated adults with classical PKU and its relationship with cognition and metabolic parameters is inconclusive. This study aims to explore the cerebral WM microstructural alterations in adult patients with early-treated classical PKU and their association with blood phenylalanine (Phe) levels and neuropsychological performance using whole-brain diffusion tensor imaging (DTI).

**Methods:**

Twenty-nine patients with early-treated classical PKU (mean age = 30.86, SD = 7.74) and 31 healthy controls (mean age = 32.45, SD = 9.40) underwent neuropsychological assessment and MRI. Phe dry blood spot (DBS-Phe) samples, along with venous Phe levels, were collected from the PKU sample to calculate the index of dietary control (IDC). Tract-based spatial statistics (TBSS) of the mean diffusivity (MD), and fractional anisotropy (FA), were carried out with FSL v6.0.4 to assess between-group differences and to explore associations with both cognitive and clinical data.

**Results:**

Patients exhibited a widespread white matter tract involvement, with lower MD and higher FA values compared to controls. The most affected tracts were the inferior longitudinal fasciculus and inferior fronto-occipital fasciculus for MD, and the anterior corona radiata, uncinate fasciculus and forceps minor for FA. MD negatively correlated with IDC and venous Phe levels, whereas FA negatively correlated with full-scale intelligence quotient (FSIQ) (*p*-value ≤0.05 FWE-corrected).

**Conclusions:**

Microstructural WM alterations were present in adults with early-treated classical PKU, and these abnormalities were related to global intelligence and metabolic control markers. Although our results suggest the importance of proper disease management, further studies are needed to determine its long-term relevance.

**Supplementary Information:**

The online version contains supplementary material available at 10.1186/s11689-025-09622-8.

## Synopsis

The article demonstrates that diffusion tensor imaging (DTI) reveals significant white matter (WM) microstructural alterations in adults with early-treated classical phenylketonuria. These alterations are associated with cognitive performance and phenylalanine levels, suggesting the importance of ongoing effective metabolic control.

## Introduction

Phenylketonuria (PKU) (OMIM #261600) is an inborn error of amino acid (AA) metabolism. This condition results from homozygous or compound heterozygous mutations in the phenylalanine hydroxylase gene (OMIM #612349), which encodes an enzyme responsible for converting phenylalanine (Phe) into tyrosine (Tyr). This induces various neuropathological outcomes, such as white matter (WM) abnormalities and neurotransmitter imbalances [1–4].

The accumulation of Phe in the brain can have toxic effects, potentially inhibiting blood-brain barrier transporters for large neutral amino acids, reducing glutamatergic synaptic transmission, decreasing cholesterol synthesis and myelin production, increasing oxidative stress, and disrupting calcium homeostasis [5]. Long-standing research has suggested a potential impairment of WM integrity in patients with PKU [6, 7]. The detrimental effects on WM arise through a complex relation between several mechanisms: initially, it is hypothesised that elevated Phe levels in the bloodstream could cross the blood-brain barrier, affecting the brain. Subsequently, this condition might significantly decrease Tyr and its derivative neurotransmitters, such as dopamine and norepinephrine, while promoting the accumulation of alternative Phe metabolites. Additionally, it may adversely affect oligodendroglia, impairing the synthesis of myelin [2–5].

Neuropathological examinations of untreated individuals revealed disrupted myelination patterns [3]. These alterations in WM are consistently observed as the most significant brain abnormalities of patients with PKU, even though many patients with early-treated PKU often perform well in daily life. The primary areas affected, which are more vulnerable to high Phe levels, include the optic tract, corpus callosum, subcortical and periventricular WM, cortico-hippocampal relay circuits, and axonal connections to the prefrontal cortex [5]. Clinical magnetic resonance imaging (MRI) techniques, such as T2-weighted and FLAIR images, specifically detect these myelination anomalies, showcasing high-signal intensity areas, mainly in the periventricular WM. In a review including 312 individuals with PKU aged between 0.9 and 49 years, Anderson and Leuzzi [3] reported abnormal WM in 93% of cases viewed on the T2 sequence.

There is substantial evidence indicating that the extent of WM abnormalities, particularly WM diffusivity, is sensitive to the metabolic management of patients [8]. In this sense, it has been reported that the severity of these abnormalities can vary according to several factors, among them dietary compliance and clinical progression [3, 9].

Diffusion tensor imaging (DTI) is considered an effective method for detecting and measuring microstructural abnormalities in WM integrity because of its ability to track diffusion [10]. It may be used to characterise the magnitude, the degree of anisotropy, and the orientation of directional diffusion. The most common DTI indices able to characterise WM integrity are the mean diffusivity (MD) and apparent diffusion coefficient (ADC), which reflect the water diffusion rate, and fractional anisotropy (FA) and radial diffusivity (RD), which reflect the asymmetry of water diffusion [11].

Previous studies with paediatric patients with PKU reported lower levels of MD [11–15]. The same results were reported in mixed-age groups of participants [7, 16, 17] and in adult samples [10, 18]. Higher ADC values were seen mostly in mixed-age samples [6, 19, 20] but also in adults [8]. In contrast, FA did not differ from controls in either paediatric samples [13] nor in mixed-age samples [6, 16, 17, 20, 21]. Positive FA findings are incongruent, as both decreases [18] and increases [7] have been observed.

Moreover, there is also a high concordance of the ageing effects of WM impairment. Mastrangelo et al. [22] conducted a longitudinal study on a large sample of age-mixed patients with PKU, utilising MRI clinical data. They found that the WM alterations showed increased severity from the second decade of life. Using quantified parameters, White et al. [11] reported that age was associated with significant differences in MD across all regions of the corpus callosum, with a significant interaction between age and group in the more anterior regions (genu, rostral body). This indicates that within the PKU group, MD became more restricted with increasing age in a group of children, unlike in the control group. Wesonga et al. [12] examined MD in relation to age across 10 brain regions of interest (ROI) in school-aged children with early- and continuously-treated PKU and found that several regions showed significant MD increments with age in PKU but not in controls. Significant group-by-age effects were seen in four regions (splenium and genu of corpus callosum, optic radiation and hippocampus) [12]. Gonzalez et al. [13], using a tract-based spatial statistics (TBSS) analysis approach in a sample of children, also found MD correlations with age. Therefore, this background underscores the necessity of investigating DTI abnormalities in adult samples while avoiding the confounding effects of maturation.

In the current study, we included a large sample of early-treated adults with classical PKU to explore the microstructural cerebral WM alterations in comparison to healthy controls and their association with cognitive performance and metabolic control indicators.

## Methods

### Participants

Participants in this study were recruited from the Muscular and Inherited Metabolic Disorders Unit, at the Hospital Clínic, located in Barcelona (Catalonia, Spain). The total sample comprised 60 participants, 29 of whom were patients with early-treated classical PKU whereas the remaining 31 participants were age and sex comparable healthy controls (HC).

The inclusion criteria for PKU patients included: (1) age above 18 years and (2) genetic diagnosis of classical PKU. The inclusion criteria for HC were age above 18 years. The exclusion criteria for patients and HC were: (1) intelligence quotient estimation below 70 according to Wechsler Adult Intelligence Scale – 4th edition (WAIS-IV) tests, (2) pregnancy or planning a pregnancy during the study period, (3) active cancer, (4) severe chronic hepatic disease, (5) acute cardiovascular event occurring within the 6 months prior to study inclusion, (6) common MRI contraindications, (7) claustrophobia, (8) pathological MRI findings other than mild WM hyperintensities in long repetition time sequences, and (9) MRI artefacts.

This study was approved by the Bioethics Committee of the University of Barcelona (IRB00003099) and Hospital Clínic of Barcelona (HCB/2020/0552). It was conducted in accordance with the basic principles of the Declaration of Helsinki, among other relevant regulations and guidelines. Signed written informed consent was provided by all the participants of this study, after a complete explanation of the procedures involved.

### Clinical data

Participants’ sociodemographic information, along with clinical data, was obtained during a clinical interview. This information included PKU date of diagnosis, previous and current pharmacological treatment (i.e. sapropterin), use of protein substitutes and supplements, Phe dry blood spot (DBS-Phe) and venous Phe levels, body mass index (BMI), subjective cognitive complaints through clinical assessment, years of education and parents’ years of education, with other medical diagnoses.

The DBS-Phe levels were measured using tandem mass spectrometry (MS/MS) using the NeoBase™ 2 Non-derivatized kit (Revvity, Inc; Waltham, Massachusetts, U.S.). Briefly, to extract the Phe from 3.2 mm of DBS, an organic compound solution that includes the deuterated Phe-d3 (internal standard) was added. Subsequently, 10 µL of this solution was directly injected into the MS/MS (Xevo-TQD; Waters Corp; Milford, Massachusetts, U.S.). The acquisition was performed in positive ionization and Multiple Reaction Monitoring modes using Masslynx software (Waters Corp). The run time was 2.5 minutes. The concentration of Phe was calculated based on the area relative to its internal standard, which had a known concentration, using Neolynx software (Waters Corp). The results were expressed in µmol/L.

Median Phe values obtained from DBS were used to determine the index of dietary control (IDC). This index was approximated using the DBS-Phe levels recorded in the year preceding the study’s inclusion, with approximately 6 to 12 measurements per year for each patient [23, 24].

For additional information on the metabolic control of the patients, historical plasma and DBS-Phe levels throughout their lifetime were collected from medical records. The median of all plasma and DBS-Phe levels recorded each year was calculated for each patient up to the day of the baseline measurement, resulting in an annual median. These annual medians were then averaged according to four age categories (childhood: 0–12 years, adolescence: 13–17 years, adulthood: ≥18 years, and lifetime), excluding age categories with fewer than 10 available measurements per patient [23, 24]. The proportion of Phe values falling within the desired range for the patient [1], categorized accordingly, was also documented. This information is detailed in Supplementary Table 1.

### Neuropsychological assessment

Participants from both groups, the HC and PKU groups, underwent a comprehensive neuropsychological assessment. This cognitive evaluation was designed based on domains typically affected in PKU, as identified in prior studies [25]. The assessment comprised several WAIS-IV subtests, including (1) the Vocabulary, (2) Similarities, (3) Arithmetic, (4) Digit Span including Forward, Backwards, and Sequencing tasks, (5) Letter-Number Sequencing, (6) Block Design, (7) Matrix Reasoning, (8) Digit-Symbol Coding, and (9) Symbol Search. Scaled scores based on normative data were obtained. From these subtests, we calculated the verbal comprehension (VCI), perceptual reasoning (PRI), working memory (WMI), and processing speed (PSI) indices (VCI and PRI, prorated) and the prorated full-Scale intelligence quotient (FSIQ) of the WAIS-IV.

### MRI acquisition

MRI were acquired with a 3 T scanner (MAGNETOM Prisma, Siemens, Germany), located at the *Centre de Diagnòstic per la Imatge de l’Hospital Clínic de Barcelona* (Catalonia, Spain). The scanning protocol of this study included two diffusion-weighted images acquired using identical parameters (TR= 3230 ms, TE= 89.20 ms, voxel size= 1.5 mm^3^, 99 diffusion directions at b= 0, 1500 and 3000 s/mm^2^, flip angle 78°, 92 slices, FOV= 210 mm; slice thickness 1.5 mm), but reversed phase-encoding direction (anterior-posterior (A-P) and posterior-anterior (P-A)), obtained for each subject. Subsequent preprocessing and analyses were performed at the Neuroimaging Laboratory of the Medical Psychology Unit, Department of Medicine, University of Barcelona, Spain.

### Tract-based spatial statistics analyses

The DTI technique was implemented to investigate local diffusion properties of white matter tracts. Specifically, FA was calculated, indicating the principal directionality of water diffusion, while MD provides information about the overall magnitude of water diffusion, both markers of WM tracts’ integrity [26]. DTI data was processed using the FSL (FMRIB Software Library, version 6.0.5) (https://fsl.fmrib.ox.ac.uk/fsl/fslwiki) [27] toolbox, developed by the Oxford Centre for Functional MRI of the Brain (FMRIB).

The preprocessing of DTI images included the application of TOPUP and EDDY correction algorithms to rectify distortions caused by eddy currents and movements, as well as to adjust for field inhomogeneities.

Following the preprocessing, we employed the DTIFIT function within FSL to fit the diffusion tensor model to each voxel. Our analysis utilised a b-value of 1500 s/mm^2^, with the imaging protocol including 14 non-diffusion-weighted volumes and 94 volumes at b= 1500 s/mm^2^, in an AP-PA acquisition.

For a group comparison and correlations with DTI metrics, our study utilised TBSS [26, 27]. TBSS performs nonlinear registration (using Nonlinear Image Registration Tool [FMRIB]) of FA images from DTIFIT to the MNI standard space and generates a mean FA skeleton that represents the centre of all WM tracts common to the whole group. Each subject’s FA image was projected onto the skeleton and the resulting FA skeleton images were fed into a general linear model (GLM) modelling the two groups (HC, PKU) to find voxel-wise differences in FA skeleton maps. The same steps were used to obtain and study the MD maps.

The Johns Hopkins University (JHU) ICBM-DTI-81 and the XTRACT HCP Probabilistic Tract atlases, both incorporated in FSLView (v3.2.0), were used to extract the anatomical labels from the FA and the MD maps. The JHU ICBM-DTI-81 atlas includes 50 white matter tract labels manually segmented from diffusion data of 81 subjects from the International Consortium of Brain Mapping. The XTRACT HCP Probabilistic Tract atlas comprises 42 white matter tracts derived from diffusion data of 178 subjects from the Human Connectome Project.

### Statistical analyses

Statistical analyses of sociodemographic, clinical, and neuropsychological data were conducted using IBM SPSS Statistics 27.0.1.0 (2020; Armonk, NY: IBM Corp) and R Statistical Software, version 4.3.1 (R Core Team 2023, https://www.r-project.org/). The Shapiro-Wilk test was employed to assess the normality of data distributions, and Levene’s test was used to evaluate the homogeneity of variances. We performed between-group comparisons for continuous variables using Student’s t-tests or Mann-Whitney U tests (for variables that did not meet the assumption of normality), depending on the data distribution. Categorical or dichotomous variables were examined using Pearson’s chi-squared test. Correlation between variables was performed with Spearman’s or Pearson’s tests when required. Results were corrected for false discovery rate (FDR) using MATLAB (v.R2020b). We set the threshold for statistical significance at *p*≤0.05.

Effect size measures were calculated to quantify the magnitude of differences between groups and the strength of relationships within our dataset.

Neuroimaging statistical analyses consisted of a voxel-wise GLM with non-parametric permutation tests (5000 permutations), besides threshold-free cluster enhancement (TFCE) for statistical inference. Voxel-wise intergroup comparisons, as well as multiple regression analyses between imaging measures, WAIS-IV indices, FSIQ, and metabolic control indicators, were computed. Only clusters >40 voxels were reported. For multiple testing corrections, the family-wise error rate (FWE) correction was applied, with a reporting criterion of FWE-corrected *p*-value ≤0.05 for all the analyses.

All the analyses were controlled for age, with the age of the participants included as a covariate.

## Results

### Sociodemographic and clinical data

No significant intergroup differences were found in age (range_HC_=19–56, range_PKU_=18–50), sex, BMI or parental education. However, there was a significant difference in years of education despite early treatment and similar educational contexts. Detailed sociodemographic and clinical information is presented in Table 1.

**Table 1:** Sociodemographic and clinical data

	N	HC	PKU^a^	Statistics	*p*-value	pFDR
Age, years	31/29	32.45±9.4	30.86±7.74	0.710^t^	0.480	0.640
Sex, female (%)	31/29	17 (54.83)	14 (48.28)	0.258^x2^	0.611	0.698
Education, years	31/29	18.00±3.15	15.00±3.72	3.377^t^	0.001	0.004
Parental education, years	31/29	11.74±3.84	10.35±4.00	1.381^t^	0.172	0.275
Subjective cognitive complaints, yes (%)	31/29	16 (51.61)	21 (72.41)	2.742^x2^	0.098	0.261
Psychiatric comorbidities	31/29	4 (12.90)	8 (27.59)	2.019^x2^	0.155	0.275
BMI (Kg/m^2^)	31/29	23.75±2.73	23.57±3.17	0.237^t^	0.814	0.814
Venous Phe levels (µmol/L)	31/29	56.20 (15.10)*	700.10 (446.70)*	0.000^U^	<0.001	<0.001
IDC – DBS Phe levels	-/29	-	720.57±323.85	-	-	-
Good control by DBS, yes (%)^b^	-/29	-	18 (62.07)	-	-	-
Good control by clinic outcome, yes (%)^c^	-/29	-	14 (48.28)	-	-	-
Sapropterin, yes (%)^d^	-/29	-	8 (27.59)	-	-	-

Values denote mean ± SD, median (IQR)*, or numbers (frequencies)

*Abbreviations: BMI* Body mass index, *DBS* dried bloodspot, *dx* diagnosed, *HC* healthy controls, *IDC* Index dietary control, *Phe* Phenylalanine, *PKU* patients with phenylketonuria

Statistical tests (*p*<0.05):^X2^Chi-Squared, ^U^Mann-Whitney, ^t^t-test

^a^The classical PKU group is formed by ‘early diagnosed’ patients (at <3 months of age) [28], 28 of them at newborn screening and one at 2 months of age

^b^According to European guidelines standards (<360µmol/L for childhood and <600µmol/L for adolescence and adulthood)^1^

^c^Phe levels remained below 600 µmol/L consistently for at least two of the past three years

^d^Sapropterin dihydrochloride (Kuvan®, BioMarin Pharmaceutical Inc.; Novato, California, U.S.), a synthetic form of the tetrahydrobiopterin (BH4), a cofactor for phenylalanine hydroxylase

Regarding current Phe levels extracted from the venous blood sample (median=700.10µmol/L, IQR=446.70), 18 of 29 participants (62.07%) had concurrent Phe levels below the limit suggested by the standards of the European guidelines for adults (600µmol/L) [1]. Moreover, attending the IDC extracted from DBS-Phe data obtained in the previous year (mean=720.57µmol/L, SD=323.85), 11 of 29 participants (37.93%) presented Phe levels below the standards. Fourteen (48.28%) participants were considered to be under good metabolic control defined as Phe levels remained below 600 µmol/L consistently for at least two of the past three years. This classification considers the long-term management patterns, to ensure accurate assessment of their Phe control status.

Historical plasma and DBS-Phe levels, categorised into different developmental stages, indicated the adjustment of the patients in metabolic control. During childhood, 7 of 17 participants (41.18%) had Phe levels above the recommended 360 µmol/L according to European guidelines. During adolescence, 5 of 24 participants (20.83%) had Phe levels above the recommended 600 µmol/L according to European guidelines. During adulthood, 12 of 26 participants (46.15%) had Phe levels above the recommended 600 µmol/L according to European guidelines. Considering the entire lifespan and including only those patients with available measurements at all developmental stages, 12 of 15 participants (80.00%) generally maintained well-adjusted Phe levels, remaining below the recommended standards of 600 µmol/L according to European guidelines for most of their lives, up to the baseline measurements of the current study.

### Neuropsychological performance

**Fig. 1:**
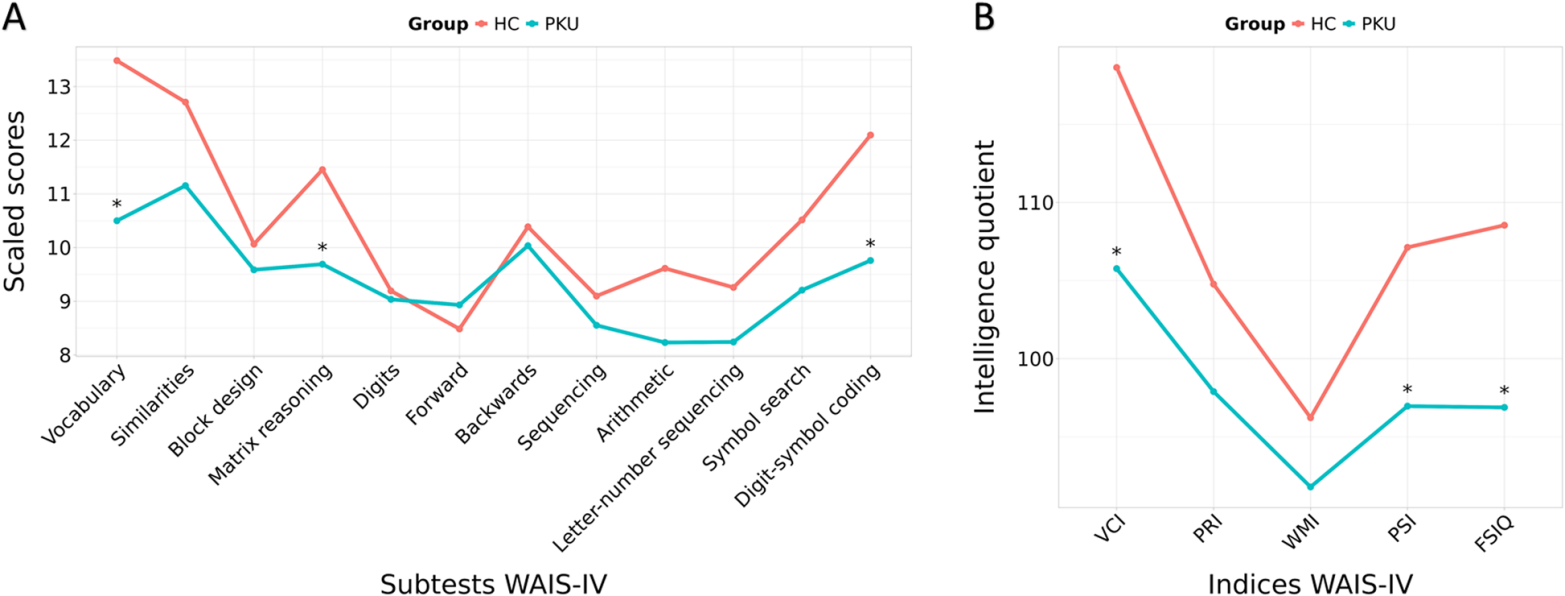
Graphic shows mean scaled scores of the subtests and indices of the WAIS-IV, per group. **A** Neuropsychological performance based on the subtests of the WAIS-IV, per group. **B** Neuropsychological performance based on the indices of the WAIS-IV, per group. Abbreviations: FSIQ, Full Scale Intelligence Quotient; HC, healthy controls; PKU, patients with phenylketonuria; PRI, Perceptual Reasoning Index; PSI, Processing Speed Index; VCI, Verbal Comprehension Index; WMI, Working Memory Index. *highlights significative results (*p*<0.05, FDR-corrected). Specific data regarding means and SD can be found in Table 2

Patients with PKU had significantly lower scores than HC in Vocabulary, Matrix reasoning, and Digit-symbol coding subtests of the WAIS-IV, in the VCI and PSI indices, and in the FSIQ (Table 2 and Fig. 1).

**Table 2:** Neuropsychological performance based on scaled scores and indices of the WAIS-IV

	N	HC	PKU	Statistics	***p***-value	pFDR	Effect size
Vocabulary	31/26	14.00 (2.00)*	9.50 (4.00)*	628.500^U^	<0.001	0.002	1.126
Similarities	31/26	12.71±2.67	11.15±2.54	2.248^t^	0.029	0.059	0.595
VCI	31/26	122.00 (17.50)*	106.00 (27.00)*	602.500^U^	0.001	0.006	0.962
Block design	31/29	10.07±2.07	9.59±2.31	0.844^t^	0.402	0.488	0.219
Matrix reasoning	31/29	11.45±2.52	9.69±2.39	2.782^t^	0.007	0.021	0.717
PRI	31/29	104.77±11.09	97.90±12.84	2.214^t^	0.031	0.059	0.575
Digits	31/29	10.00 (2.50)*	9.00 (3.00)*	473.500^U^	0.725	0.725	0.064
*Forward*	31/29	8.48±2.84	8.93±2.39	−0.662^t^	0.511	0.579	0.170
*Backwards*	31/29	10.39±3.06	10.03±2.13	0.521^t^	0.605	0.643	0.133
*Sequencing*	31/29	9.10±2.40	8.55±2.47	0.866^t^	0.390	0.488	0.224
Arithmetic	31/26	9.61±2.67	8.23±2.73	1.923^t^	0.060	0.102	0.513
Letter-number sequencing	31/29	9.26±2.82	8.24±2.49	1.484^t^	0.143	0.221	0.382
WMI	31/29	96.23±12.20	91.79±12.75	1.374^t^	0.175	0.248	0.356
Symbol search	31/29	10.52±1.79	9.21±2.46	2.349^t^	0.023	0.055	0.613
Digit-symbol coding	31/29	12.10±2.82	9.76±2.67	3.300^t^	0.002	0.006	0.851
PSI	31/29	107.13±10.92	96.97±11.55	3.496^t^	0.001	0.005	0.905
FSIQ	31/26	108.55±10.41	96.88±11.64	3.953^t^	<0.001	0.002	1.062

Values denote the mean ± SD, or median (IQR)* of scaled scores of the subtests and corrected indices of WAIS-IV

Visual information of the data can be found in Fig. 1

*Abbreviations: FSIQ* Full Scale IQ, *HC* healthy controls, *PKU* patients with phenylketonuria, *PRI* Perceptual Reasoning Index, *PSI* Processing Speed Index, *VCI* Verbal Comprehension Index, *WAIS-IV* Wechsler Adult Intelligence Scale IV, *WMI* Working Memory Index

Statistical tests used: ^U^Mann-Whitney, ^t^t-test(*p*<0.05)

The performance in the Similarities and Symbol-search subtests of the WAIS-IV, as well as in the PRI, was poorer in patients with PKU compared to HC. However, these differences did not survive FDR correction.

The correlation between neuropsychological variables and metabolic control markers in PKU was investigated, but no significant associations were found.

### DTI whole-brain comparison between groups

**Fig. 2:**
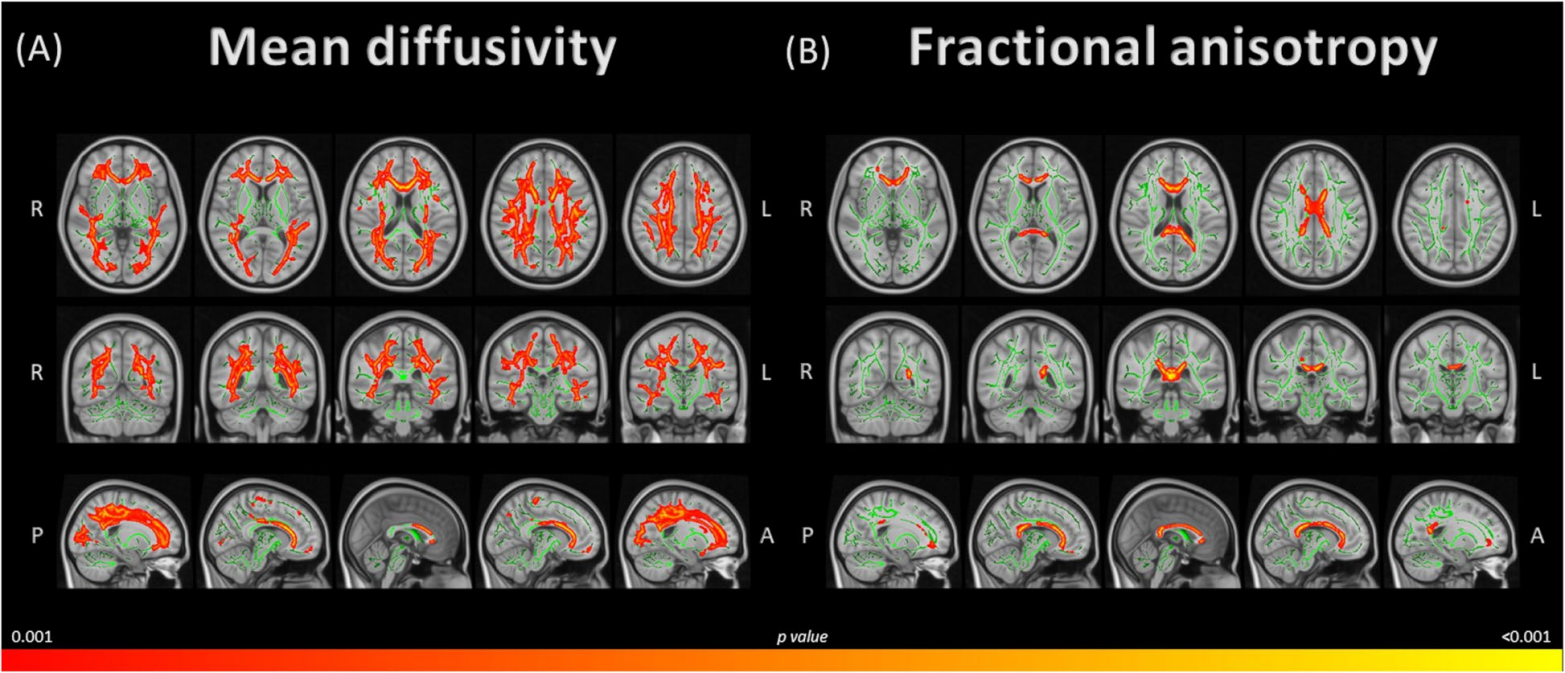
Group differences in whole-brain MD and FA. **A** Lower MD values at whole-brain in the PKU group vs the HC group with age as a covariate. **B** Higher FA values at whole-brain in the PKU group vs the HC group with age as a covariate. Differences between groups are calculated through tract-based spatial statistics (TBSS). Results are overlaid on the WM skeleton (green) and displayed over the sagittal and axial sections of the MNI 152 standard brain, at *p*<0.001 TFCE-corrected. Specific data regarding highest statistically significant voxel, clusters and tracts can be found in Table 3, and other MD and FA significant tracts in Supplementary Table 2 and Supplementary Table 3, respectively. Abbreviations: A, anterior; L, left; P, posterior; PKU, patients with phenylketonuria; R, right

Compared to the HC group, patients with PKU showed lower levels of MD, with the highest significance in the inferior longitudinal fasciculus and the inferior fronto-occipital fasciculus (Table 3, Fig. 2A). Significant results were extended to other tracts listed in Supplementary Table 2.

**Table 3:** Significant clusters showing MD and FA differences between groups (PKU vs HC), age-controlled

Cluster	Voxels	*p*-value (maximum)	*x*, *y*, *z* coordinates			H	Anatomical labels (maximum)
MD PKU group < HC group							
1	78863	<0.001	−41	−12	−20	Left	Inferior longitudinal fasciculus^b^/Inferior fronto-occipital fasciculus^b^
FA PKU group > HC group							
1	39014	<0.001	12	29	−10	Right	Anterior corona radiata^a^/Uncinate fasciculus^b^/Forceps minor^b^
2	269	0.047	47	−45	−1	Right	Arcuate fasciculus^b^/Inferior longitudinal fasciculus^b^
3	209	0.049	−19	17	41	Left	Frontal aslant tract^b^/Superior longitudinal fasciculus^b^
4	103	0.049	−27	−61	32	Left	Superior longitudinal fasciculus^a,b^

Coordinates of the maximum intensity voxel, given as *x*, *y*, *z* in standard space coordinates (mm)

Table shows significant clusters (*p*≤ 0.05, TFCE-corrected)

*Abbreviations: FA* fractional anisotropy, *H* Hemisphere, *HC* healthy controls, *MD* mean diffusivity, *PKU* patients with phenylketonuria

Anatomical labels were extracted from the ^a^Johns Hopkins University (JHU) ICBM-DTI-81 and the ^b^XTRACT HCP Probabilistic Tract atlases incorporated in the FSLView (3.2.0)

Higher levels of FA were found in PKU compared to the HC group (Table 3, Fig. 2B), with the highest significance located in the anterior corona radiata, uncinate fasciculus and forceps minor tracts. Significant results were extended to other tracts listed in Supplementary Table 3.

### Correlations between DTI whole-brain maps and neuropsychological performance

**Fig. 3:**
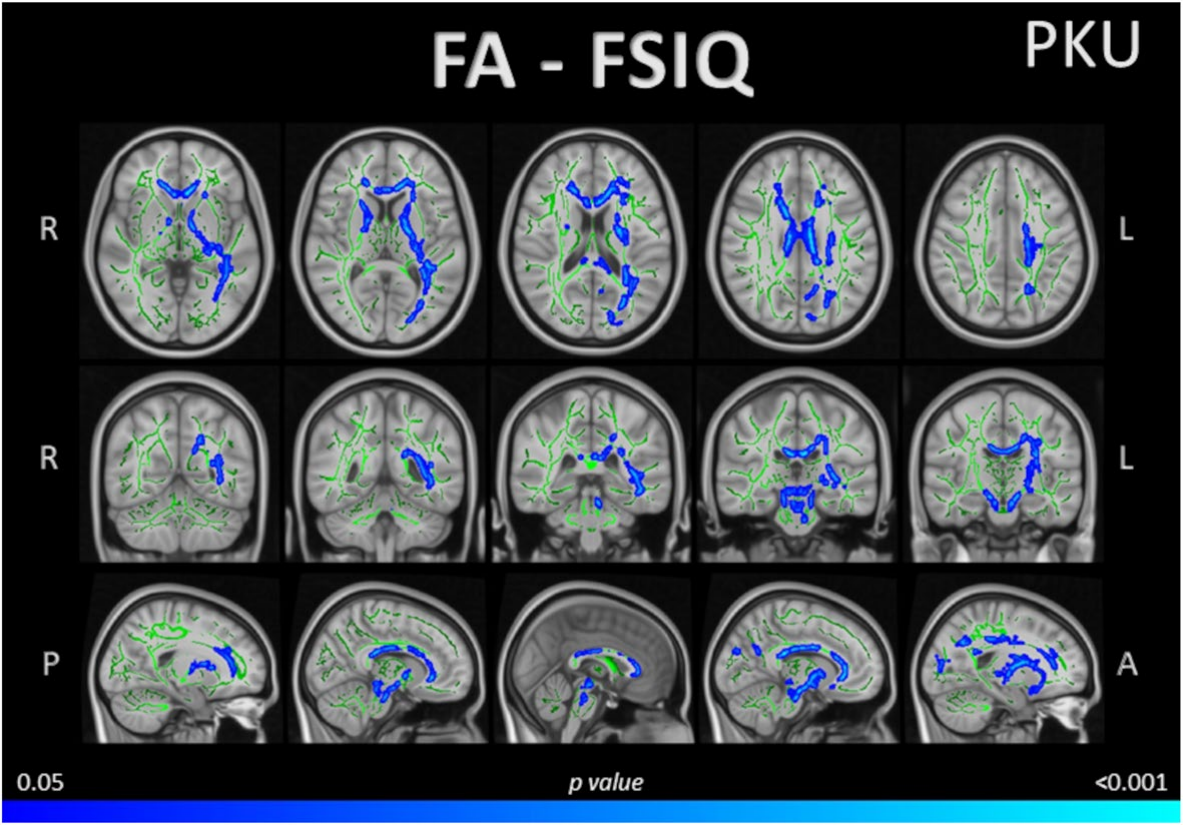
Whole-brain tract-based correlation between FA and FSIQ, in the PKU group. The image shows a negative correlation between whole-brain FA and the PKU group’s FSIQ performance with age as a covariate. Results are overlaid in white matter skeleton tracts (green), through tract-based spatial statistics (TBSS) and displayed over the axial, coronal, and sagittal sections of the MNI 152 standard brain (*p*<0.05, TFCE-corrected). Significant negative correlations are marked in cold colours. Specific data is detailed in Table 4. Abbreviations: A, anterior; FA, fractional anisotropy; FSIQ, Full Scale IQ; L, left; P, posterior; PKU, patients with phenylketonuria; R, right

Correlation analyses were performed between MD and FA maps and WAIS-IV indices that remained significant in the group comparison (VCI, PSI) and FSIQ. In the PKU group, there was a negative correlation between whole-brain FA and FSIQ, primarily in the forceps minor, longitudinal fasciculus, corticospinal tract, corpus callosum, thalamic radiation, internal capsule, cerebral peduncle, and inferior fronto-occipital fasciculus (Table 4). This correlation extended through the uncinate fasciculus, frontal aslant tract, corona radiata, fornix, anterior commissure, cingulum, sagittal stratum, external capsule, arcuate fasciculus, acoustic radiation, tapetum, forceps major, optic radiation, and vertical occipital fasciculus (Fig. 3).

**Table 4:** Significant clusters showing correlation between FA and FSIQ age-controlled, in the PKU group

Cluster	Voxels	*p*-value (maximum)	*x*, *y*, *z* coordinates			H	Anatomical labels (maximum)
Negative correlation between FA and FSIQ (WAIS-IV)							
1	8863	0.028	−10	32	5	Left	Genu of corpus callosum^a^/Forceps minor^b^
2	1109	0.039	−7	−31	21	Left	Splenium of corpus callosum^a^
3	630	0.042	13	−25	−18	Right	Cerebral peduncle^a^/Corticospinal tract^b^
4	223	0.045	18	11	6	Right	Anterior limb of internal capsule^a^/Anterior thalamic radiation^b^
5	182	0.048	−17	−23	35	Left	Body of corpus callosum^a^/Superior longitudinal fasciculus^b^
6	82	0.050	9	−22	−26	Right	Corticospinal tract^a,b^
7	56	0.050	18	−7	5	Right	Posterior limb of internal capsule^a^/Superior thalamic radiation^b^

Coordinates of the maximum intensity voxel, given as *x*, *y*, *z* in standard space coordinates (mm). Table shows significant clusters >40 voxels (*p*≤ 0.05, TFCE-corrected)

Visual detailed information at Fig. 3

*Abbreviations*: *FA* fractional anisotropy, *FSIQ* Full Scale IQ, *H* Hemisphere, *WAIS-IV* Wechsler Adult Intelligence Scale IV

Anatomical labels were extracted from the ^a^Johns Hopkins University (JHU) ICBM-DTI-81 and the ^b^XTRACT HCP Probabilistic Tract atlases incorporated in the FSLView (3.2.0)

No correlation was found between MD and FSIQ.

### Correlations between DTI whole-brain maps and metabolic control markers

**Fig. 4:**
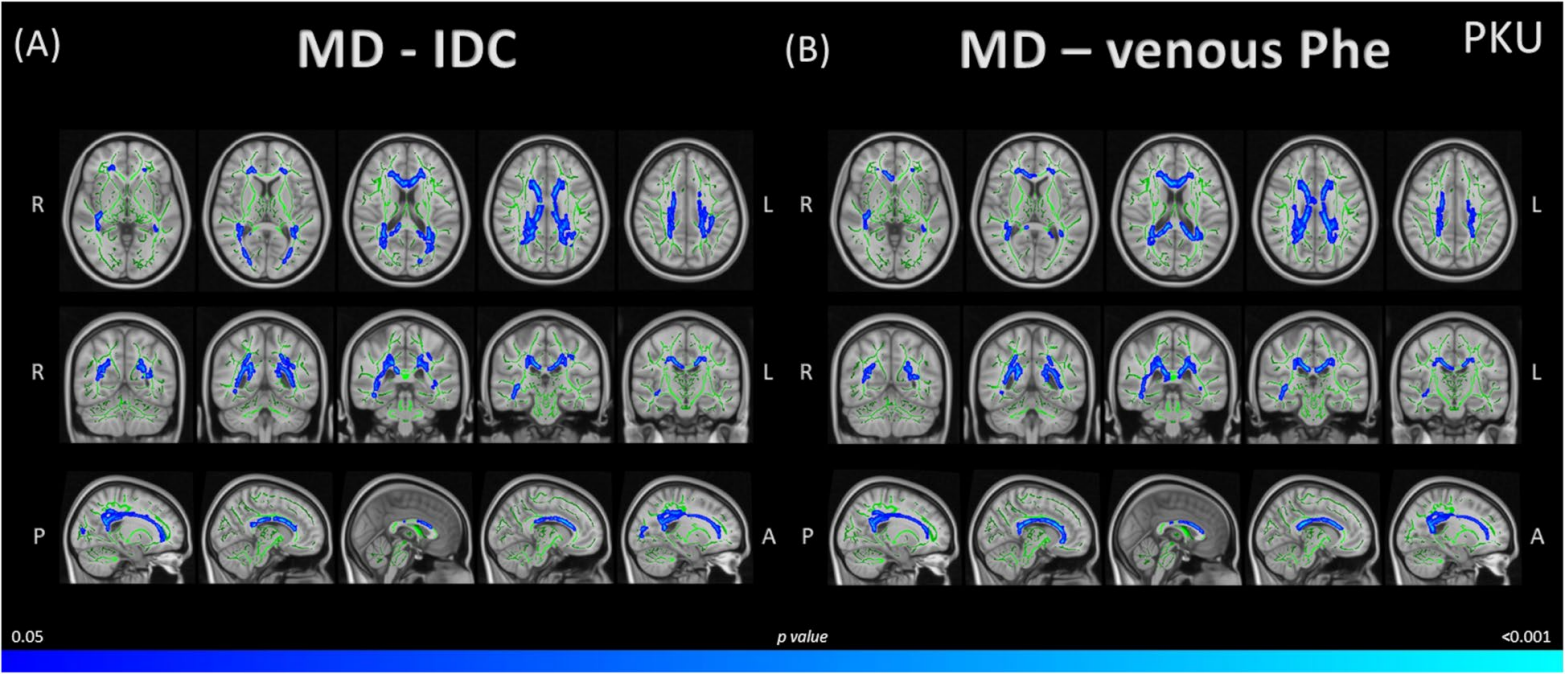
Correlations between MD and Phe levels in the PKU group. **A** Significant negative correlation between MD and IDC Phe levels in PKU with age as a covariate. **B** Significant negative correlation between MD and venous Phe levels in PKU with age as a covariate. Results are overlaid in white matter skeleton tracts (green), through tract-based spatial statistics (TBSS), and displayed over the sagittal, coronal, and axial sections of the MNI 152 standard brain (*p*<0.05, TFCE-corrected). Significant negative correlations are marked in cold colours. Abbreviations: A, anterior; IDC, index dietary control; L, left; MD, mean diffusivity; P, posterior; Phe, Phenylalanine; PKU, patients with phenylketonuria; R, right. Specific data is detailed in Table 5

IDC negatively correlated with MD, primarily in the corpus callosum and longitudinal fasciculus (Table 5). This correlation extended through the uncinate fasciculus, forceps minor and major, frontal aslant tract, inferior fronto-occipital fasciculus, corona radiata, arcuate fasciculus, thalamic radiation, corticospinal tract, acoustic radiation, sagittal stratum, external capsule, tapetum, optic radiation, and vertical occipital fasciculus (Fig. 4A).

**Table 5:** Correlations between MD with IDC and venous Phe levels age-controlled, in the PKU group

Cluster	Voxels	*p*-value (maximum)	*x*, *y*, *z* coordinates			H	Anatomical labels (maximum)
Negative correlation between MD and IDC Phe levels							
1	8370	0.018	17	−28	30	Right	Body of corpus callosum^a^
2	161	0.047	−33	−31	37	Left	Superior longitudinal fasciculus^a,b^
Negative correlation between MD and venous Phe levels							
1	7298	0.026	14	−23	29	Right	Body of corpus callosum^a^
2	431	0.048	42	−22	−11	Right	Sagittal stratum^a^/Inferior fronto-occipital fasciculus^b^/Inferior longitudinal fasciculus^b^

Coordinates of the maximum intensity voxel, given as *x*, *y*, *z* in standard space coordinates (mm). The table shows significant clusters >40 voxels (p≤ 0.05, TFCE-corrected)

*Abbreviations: H* Hemisphere, *IDC* Index of Dietary control, *MD* mean diffusivity, *Phe* Phenylalanine, *PKU* patients with phenylketonuria

Anatomical labels were extracted from the ^a^Johns Hopkins University (JHU) ICBM-DTI-81 and the ^b^XTRACT HCP Probabilistic Tract atlases incorporated in the FSLView (3.2.0)

Similarly, venous Phe levels negatively correlated with MD values, primarily in the corpus callosum, longitudinal fasciculus, sagittal stratum, and inferior fronto-occipital fasciculus (Table 5). This correlation extended through the uncinate fasciculus, forceps minor and major, frontal aslant tract, corona radiata, cingulum, arcuate fasciculus, thalamic radiation, corticospinal tract, acoustic radiation, external capsule, tapetum, and optic radiation (Fig. 4B).

There was no correlation between the FA map and IDC or venous Phe levels.

## Discussion

In our study, adult patients with early-treated classical PKU differed from HC in DTI parameters across various WM tracts. These DTI differences were related to Phe levels and cognitive functions of the patients with classical PKU.

We observed reduced MD values across multiple brain regions, aligning with findings from previous studies of paediatric, mixed [7, 11–17] and adult samples [10, 18]. This consistency reinforces the validity of our results.

Moreover, whole-brain analysis revealed higher FA values, corroborating the findings from Hawks et al. [7]. Thus, this suggests that the integrity of fibres is not compromised. In patients with evident WM damage of fibres, such as those with multiple sclerosis [29] or traumatic brain injury [30], there are clear reductions in FA accompanied by increases in MD, which is the inverse pattern observed in patients with PKU. Probably, lower MD and higher FA values may be attributed to water accumulation within myelin sheaths, indicating intramyelinic swelling without significant myelin loss, as suggested by Anderson and Leuzzi [3]. These authors supported their hypotheses in a previous histopathological study, which suggests the impairment of previously assembled myelin in treated patients [9]. The reversal of white matter abnormalities in early-treated patients with PKU [31, 32] may indicate the previous existence of intramyelinic edema, characterized by the swelling of myelin sheaths [8]. This separation could reduce MD and increase FA due to water restrictions. However, to the best of our knowledge, no previous studies have confirmed the association between diffusivity changes and the histopathological abnormalities reported in PKU.

Contrary to our results, Muri et al. [18] studied DTI metrics in adult patients with early-treated classical PKU and found lower FA compared to HC. While the sociodemographic data and analyses are comparable between their study and ours, their patient cohort has a slightly higher level of education compared to HC, and their HC sample size is somewhat larger than ours. Additionally, there are differences in the MRI acquisition protocols used in both studies. Since findings regarding FA are still controversial, further multicentre studies with larger samples are needed.

The clinical significance of WM alterations observed with DTI has been a subject of debate. DTI is an excellent technique for observing subtle abnormalities in WM integrity; however, these abnormalities can sometimes be clinically insignificant. In this sense, it is important to correlate DTI parameters with clinical data. In contrast to previous studies [11–13, 17, 33], we did not find correlations with age in patients with PKU. The reason could lie in the fact that the age effect observed in previous studies may be attributed to the maturation of WM during childhood and early adulthood.

Importantly, our whole-brain analysis identified that MD values correlated with both Phe levels measurements (IDC and venous Phe) in key regions, including the superior longitudinal fasciculus, anterior, superior and posterior corona radiata, sagittal stratum, genu, body and splenium of corpus callosum, consistent with the findings of Peng et al. [21]. These correlations highlight the detrimental effects of Phe on WM, reinforcing the need for effective Phe level management. The influence of Phe levels on WM structure is well-documented [8]. It has been suggested that high Phe concentrations impair dendritic pruning and synaptic remodelling, thereby reducing neurocognitive performance [5]. Nevertheless, a recent study suggested that, despite the adverse effects of high Phe levels on WM structure, these changes could be reversible with an appropriate Phe level management following short-term high Phe exposure [32].

Neuropsychological assessment showed significantly lower performance in the PKU group compared to HC in several WAIS-IV subtests and indices. The most compromised cognitive functions in between-group comparisons were word knowledge and verbal concept formation, and processing speed, assessed using the Vocabulary and Symbol-digit coding subtests, respectively, as well as the global intelligence quotient, assessed using the FSIQ, all of which showed high effect sizes. Although the neuropsychological performance was lower in the PKU group than in HC, the scores remained within the normative range, highlighting the success of PKU treatment compared to other rare metabolic disorders. Of note, all patients in the PKU group had classical PKU and received early and continuous treatment. Differences in years of education between groups could have influenced IQ performance, particularly in the Vocabulary subtest and VCI, but are likely to have less impact on subtests like Digit-Symbol Coding. The differences in IQ scores between patients with PKU and HC are a consistent finding. Although the IQ scores of adults with PKU were within normal range, they were consistently lower compared to HC [34]. In a meta-analysis comparing 407 patients with PKU with 439 HC, an effect size of 0.60 for IQ was reported [25], whereas we obtained a higher effect size. Differences in IQ between patients with PKU and HC could also be attributed to the recruitment of the HC group. Despite both groups having similar sociocultural backgrounds, it is possible that individuals with higher IQs were more likely to volunteer, thus influencing the results.

Few previous DTI studies have correlated WM differences with cognitive performance, and the reported evidence showed controversial results. Muri et al. [18] found correlations between decreased DTI metrics (FA and MD) and lower performance in attention and cognitive flexibility tasks in adult early-treated patients with PKU using a ROIs approach. However, no whole-brain correlation analyses were reported. Regarding previous studies with pediatric [11, 13] or mixed samples [21, 33], only one study found lower MD in the corpus callosum associated with poorer verbal fluency [33]. This study included individuals aged 6 to 35 and accounted for age in the ROI analyses. Additionally, there were other studies focused on the relationship between WM structure and cognition that did not use DTI techniques. Anderson et al. [35], reported that children with extensive WM alterations, as assessed using MRI clinical data, had decreased performance in attention and executive functions. In adults, Nardecchia et al. [36] did not find correlations between intelligence quotient or executive functions and white matter alterations. Weglage et al. [37], in a five-year follow-up, claimed that despite the persistence of neuropsychological impairment, cognitive performance was unrelated to MRI findings.

Notably, our findings revealed a significant correlation between the FA map and FSIQ. This correlation is particularly relevant in long fascicles linking associative cortical regions involved in complex cognitive functions such as the longitudinal and inferior fronto-occipital fasciculus, corticospinal tract, optic and thalamic radiation, corona radiata, sagittal stratum, forceps minor, as well as the corpus callosum. The relevance of these tracts in PKU patients is reinforced by the findings arising from group comparisons and also with the results obtained in the correlations between MRI and Phe levels. Higher cerebral Phe levels have been associated with poorer cognitive functioning in early-treated adults [38]. Nevertheless, our results did not confirm the association between metabolic control and cognitive performance.

The strengths of our study lie in the inclusion of a relatively large sample of adults with early-treated classical PKU and comparable HC, along with detailed clinical, neuropsychological, and neuroimaging evaluations. We performed whole-brain analyses, and all the results were corrected for multiple comparisons. This method enabled us to identify widespread alterations in WM tracts, critical for efficient neural communication and overall cognitive function.

However, limitations should also be acknowledged. First, although our cohort was more homogeneous compared to the samples of previous studies in the field, 28 individuals were diagnosed with newborn screening, and one of them was diagnosed at 2 months of age, so dietary treatment was initiated later for this one. However, as patients with an estimated intelligence quotient <70 (WAIS-IV) were excluded, it is unlikely that our results were subjected to significant bias due to sample inclusion criteria. Second, while classically the IDC is calculated as the median of Phe levels and the mean of all medians throughout the period of interest (childhood, adolescence, adulthood) [24], we focused on the median of DBS-Phe levels in the first year before the inclusion. This limitation arose because while paediatric history records were accessible for 17 participants, data were not available for all participants. As the calculated first-year IDC allows the standardization of the same measure for the whole group and corrects for sporadic decompensations (e.g., illness, dietary abandonment), the selected approach offers a good picture of adults’ metabolic control. Third, some of our patients were receiving sapropterin treatment, which could potentially influence our results. However, the sample size of patients under sapropterin treatment was insufficient to conduct a more detailed investigation into this effect. Finally, educational differences between groups could have influenced FSIQ scores.

In conclusion, despite early treatment, adult patients with classical PKU exhibit WM microstructural abnormalities and poorer cognitive performance in comparison to HC. The observed pattern of lower MD and higher FA contrasts with that seen in patients suffering from WM damage, such as in multiple sclerosis and traumatic brain injury. Crucially, lower MD values correlate with Phe levels, while higher FA values correlate with global cognition scores. This intricate relationship suggests the importance of metabolic control in maintaining normal WM structure and highlights the impact of PKU on cognitive performance.

## Supplementary Information

Supplementary Material 1.

## Data Availability

The datasets generated and/or analysed during the current study are not publicly available due to privacy concerns for the research participants but are available from the corresponding author upon reasonable request.

## Abbreviations

AA: Amino acid
ADC: Apparent diffusion coefficient
A-P: Anterior-posterior
BMI: Body mass index
DBS: Dry blood spot
DBS-Phe: Phenylalanine dry blood spot
DTI: Diffusion tensor imaging
FA: Fractional anisotropy
FDR: False discovery rate
FSIQ: Full-scale intelligence quotient
FWE: Family-wise error
GLM: General linear model
HC: Healthy controls
IDC: Index of dietary control
MD: Mean diffusivity
MRI: Magnetic resonance imaging
MS/MS: Mass spectrometry
P-A: Posterior-anterior
Phe: Phenylalanine
PKU: Phenylketonuria
PRI: Perceptual reasoning index
PSI: Processing speed index
RD: Radial diffusivity
ROI: Regions of interest
TBSS: Tract-based spatial statistics
TFCE: Threshold-free cluster enhancement
Tyr: Tyrosine
VCI: Verbal comprehension index
WAIS-IV: Wechsler Adult Intelligence Scale 4th edition
WM: White matter
WMI: Working memory index
