# Supplementary material for: Diffusivity alterations related to cognitive performance and phenylalanine levels in early-treated adults with phenylketonuria
Source: J Neurodev Disord. 2025 Jul 2;17:37. doi: 10.1186/s11689-025-09622-8 (PMC12220134; doi:10.1186/s11689-025-09622-8)
Supplement: Supplementary file 1 — Supplementary Material 1. [file 11689_2025_9622_MOESM1_ESM.docx]

**Supplementary Table 1.** Mean levels of phenylalanine and percentage of Phenylalanine measurements above the recommended standards per patient during childhood (≤12 years old), adolescence (13-17 years old), adulthood (≥18 years old), the present study, and lifetime.

| Code | Childhood | | Adolescence | | Adulthood | | Present study* | | Lifetime | |
| --- | --- | --- | --- | --- | --- | --- | --- | --- | --- | --- |
|  | Mean | % | Mean | % | Mean | % | Mean | % | Mean | % |
| A01 | - | - | 483 | 21.82 | 408 | 7.45 | 380 | 0.00 | - | - |
| A02 | - | - | 639 | 56.67 | 472 | 14.02 | 416 | 0.00 | - | - |
| A03 | 252 | 19.23 | 277 | 7.27 | 316 | 2.92 | 277 | 0.00 | 292 | 7.90 |
| A04 | - | - | 513 | 21.95 | 600 | 51.38 | 512 | 0.00 | - | - |
| A05 | 317 | 38.74 | 425 | 13.73 | 580 | 42.86 | 701 | 100.00 | 398 | 35.24 |
| A06 | - | - | 438 | 20.31 | 533 | 18.72 | 621 | 100.00 | - | - |
| A07 | - | - | 564 | 41.38 | 1011 | 96.67 | 741 | 100.00 | - | - |
| A08 | - | - | - | - | 329 | 7.69 | 513 | 0.00 | - | - |
| A09 | 237 | 11.23 | 317 | 0.00 | 385 | 3.68 | 334 | 0.00 | 323 | 6.84 |
| A11 | 523 | 88.24 | 773 | 82.00 | 992 | 93.02 | 980 | 100.00 | 895 | 87.27 |
| A14 | 322 | 45.24 | 518 | 20.34 | 660 | 37.59 | 449 | 0.00 | 542 | 37.42 |
| A15 | 416 | 54.48 | 663 | 64.71 | 522 | 21.05 | 624 | 100.00 | 482 | 48.68 |
| A16 | 296 | 24.11 | 357 | 0.00 | - | - | 277 | 0.00 | - | - |
| A18 | - | - | - | - | 720 | 85.71 | 733 | 100.00 | - | - |
| A20 | 360 | 58.12 | 589 | 50.00 | 756 | 75.00 | 576 | 0.00 | 582 | 61.02 |
| A23 | 404 | 61.29 | 458 | 22.45 | 462 | 12.88 | 299 | 0.00 | 448 | 27.16 |
| A24 | 777 | 97.56 | 721 | 71.43 | 988 | 93.55 | 981 | 100.00 | 870 | 88.17 |
| A25 | 244 | 23.45 | 505 | 24.53 | 918 | 71.88 | 628 | 100.00 | 517 | 27.55 |
| A27 | 515 | 88.41 | 602 | 40.35 | 708 | 68.83 | 921 | 100.00 | 618 | 72.79 |
| A29 | - | - | 573 | 46.43 | 710 | 64.41 | 1000 | 100.00 | - | - |
| A30 | 323 | 39.47 | 435 | 9.80 | 562 | 38.38 | 551 | 0.00 | 433 | 33.70 |
| A32 | - | - | - | - | 431 | 11.95 | 138 | 0.00 | - | - |
| A33 | 424 | 62.75 | 537 | 39.02 | 710 | 76.00 | 539 | 0.00 | 589 | 61.14 |
| A34 | 302 | 35.04 | 314 | 0.00 | 476 | 17.11 | 485 | 0.00 | 395 | 27.98 |
| A35 | - | - | 411 | 21.74 | 529 | 24.11 | 473 | 0.00 | - | - |
| A37 | - | - | - | - | - | - | 300 | 0.00 | - | - |
| A45 | 352 | 49.58 | 335 | 11.43 | - | - | 371 | 0.00 | - | - |
| A47 | 289 | 35.66 | 339 | 5.71 | 588 | 38.00 | 426 | 0.00 | 383 | 33.05 |
| A48 | - | - | - | - | 819 | 93.75 | 731 | 100.00 | - | - |

Mean phenylalanine levels measured from dry blood spot (DBS) or plasma are expressed in µmol/L. The percentages represent the proportion of phenylalanine measurements above the recommended standards within the age range category for patient according to European guidelines (360µmol/L during childhood and 600µmol/L during adolescence and adulthood)^1^.

**Supplementary Table 2** Significant tracts in between-group whole-brain analysis in the MD map

| **Category** | **Tract name** | **Abbreviation** |
| --- | --- | --- |
| *Projection fibers* | Acoustic radiation^2^ | AR |
|  | Anterior corona radiata^1^ | ACR |
|  | Anterior thalamic radiation^2^ | ATR |
|  | Corticospinal tract^2^ | CST |
|  | Optic radiation^2^ | OR |
|  | Posterior corona radiata^1^ | PCR |
|  | Posterior limb of internal capsule^1^ | PLIC |
|  | Posterior thalamic radiation^1^ | PTR |
|  | Retrolenticular part of the internal capsule^1^ | RLIC |
|  | Superior corona radiata^1^ | SCR |
|  | Superior thalamic radiation^2^ | STR |
| *Commissural fibers* | Anterior commissure^2^ | AC |
|  | Body of the corpus callosum^1^ | BCC |
|  | Forceps major^2^ | FMA |
|  | Forceps minor^2^ | FMI |
|  | Genu of corpus callosum^1^ | GCC |
|  | Splenium of corpus callosum^1^ | SCC |
|  | Tapetum^1^ | TAP |
| *Association and/or limbic fibers* | Arcuate fasciculus^2^ | AF |
|  | Cingulum (cingulate gyrus)^1^ | CgC |
|  | Cingulum subsection: Dorsal^2^ | CBD |
|  | Cingulum subsection: Peri-genual^2^ | CBP |
|  | Cingulum subsection: Temporal^2^ | CBT |
|  | External capsule^1^ | EC |
|  | Fornix^1,2^ | FX |
|  | Frontal aslant tract^2^ | FA |
|  | Inferior fronto-occipital fasciculus^2^ | IFO |
|  | Inferior longitudinal fasciculus^2^ | ILF |
|  | Middle longitudinal fasciculus^2^ | MdLF |
|  | Superior longitudinal fasciculus^1,2^ | SLF |
|  | Sagittal stratum^1^ | SS |
|  | Uncinate fasciculus^2^ | UF |
|  | Vertical occipital fasciculus^2^ | VOF |

Anatomical labels are extracted from the ^1^Johns Hopkins University (JHU) ICBM-DTI-81 and the ^2^XTRACT HCP Probabilistic Tract atlases incorporated in the FSLView (3.2.0)

**Supplementary Table 3** Significant tracts in between-group whole-brain analysis in the FA map

| **Category** | **Tract name** | **Abbreviation** |
| --- | --- | --- |
| *Projection fibers* | Anterior corona radiata^1^ | ACR |
|  | Anterior thalamic radiation^2^ | ATR |
|  | Posterior thalamic radiation^1^ | PTR |
|  | Superior corona radiata^1^ | SCR |
| *Commissural fibers* | Body of corpus callosum^1^ | BCC |
|  | Forceps major^2^ | FMA |
|  | Forceps minor^2^ | FMI |
|  | Genu of corpus callosum^1^ | GCC |
|  | Splenium of corpus callosum^1^ | SCC |
| *Association and/or limbic fibers* | Cingulum (cingulate gyrus)^1^ | CgC |
|  | Cingulum subsection: Dorsal^2^ | CBD |
|  | Cingulum subsection: Peri-genual^2^ | CBP |
|  | Inferior fronto-occipital fasciculus^2^ | IFO |
|  | Uncinate fasciculus^2^ | UF |

Anatomical labels were extracted from the ^1^Johns Hopkins University (JHU) ICBM-DTI-81 and the ^2^XTRACT HCP Probabilistic Tract atlases incorporated in the FSLView (3.2.0)
